# Sharing Experiences from the Field: updates from the Nigeria Field Epidemiology and Laboratory Training Program

**DOI:** 10.11604/pamj.supp.2019.32.1.18136

**Published:** 2019-01-21

**Authors:** Patrick Mboya Nguku, Chukwuma David Umeokonkwo, Muhammad Shakir Balogun, Ndadilnasiya Endie Waziri, Adebobola Toluwalashe Bashorun, Godwin Ntadom, Chikwe Ihekweazu

**Affiliations:** 1 African Field Epidemiology Network 50 Haile Selassie Asokoro Abuja, Nigeria; 2 Federal Ministry of Health, New Federal Secretariat Complex, Phase III, Ahmadu Bello Way, Central Business District, FCT Abuja, Nigeria; 3 Nigeria Centre for Disease Control, Plot 801, EbituUkiwe Street, Jabi, Abuja, Nigeria

**Keywords:** Field Epidemiology and Laboratory Training Programs, Nigeria, epidemiologists

## Abstract

Field Epidemiology and Laboratory Training Programs (FELTP) or Applied Epidemiology Training Programs (AETP) is based on the philosophy of “learning while doing” and application of epidemiology methods to improve public health and health care [1]. Trainees or residents in FELTP are required to conduct field investigations, data analysis, surveillance evaluations and other field-based activities while being mentored by experienced epidemiologists. Residents' work is not completed until they have shared their unique field experiences, findings and recommendations with relevant public health authorities for action. Additionally, publishing their field experiences and evidence based public health actions ensures that these training experiences are shared with the wider scientific and public health audience. In this second supplement from the Nigeria Field Epidemiology and Laboratory Training Program (NFELTP), we present investigations and studies carried out by these early career epidemiologists as a way of disseminating important public health findings [2, 3]. The supplement covers summaries of a surveillance system evaluation, secondary data analyses and several protocol-based studies. The output represents the product of field-based interactions that the residents had in the course of their experiential training in applied epidemiology. This builds up on the first NFELTP supplement published in July 2014 [3] and the progress that the program has made since its inception in 2008. This collection of articles comprises a wide variety of subjects ranging from infectious disease epidemiology (malaria, HIV, measles, rubella, tuberculosis, Ebola virus disease, pertussis) to non-communicable diseases and injuries (road traffic crashes, intimate partner violence). It also represents the work carried out in nine states and the Federal Capital Territory in Nigeria (Abuja, Anambra, Enugu, Gombe, Kano, Kaduna, Niger, Ogun & Ondo) and Sierra Leone. Most of the studies involving HIV, tuberculosis and malaria were conducted in response to national priorities set by stakeholders in Nigeria [4]. This demonstrates the integration of the FETP in the national health system and ensures that it continues to be relevant in protecting the health of the populace. The NFELTP is part of the newly created Nigeria Centre for Disease Control, the country's National Public Health Institute [5]. We expect that these articles will stimulate further discussion and help to identify other relevant research questions to be addressed across Nigeria and the African region on these important public health issues. We also hope that the wider public health audience will find in them the needed information to provide solutions to various public health challenges. We appreciate the leadership the Federal Ministry of Health in supporting the ongoing training and utilization of field epidemiologists through the NFELTP. We also acknowledge the funding and technical support provided by the African Field Epidemiology Network and the US Centers for Disease Control and Prevention.

## Competing interest

The authors declare no competing interest.
